# Quantification of the CM-Dil-labeled human umbilical cord mesenchymal stem cells migrated to the dual injured uterus in SD rat

**DOI:** 10.1186/s13287-020-01806-4

**Published:** 2020-07-13

**Authors:** Jia-Hua Zheng, Jing-Kun Zhang, De-Sheng Kong, Yan-Biao Song, Shuang-Dan Zhao, Wen-Bo Qi, Ya-Nan Li, Ming-le Zhang, Xiang-Hua Huang

**Affiliations:** 1 Department of Gynecology, The Second Hospital of Hebei Medical University, Shijiazhuang, Hebei China; 2 Department of Central Laboratory, The Second Hospital of Hebei Medical University, Shijiazhuang, Hebei China

**Keywords:** CM-Dil, Differentiate, Human umbilical cord mesenchymal stem cells, Intrauterine adhesions, Quantification

## Abstract

**Background:**

Human umbilical cord mesenchymal stem cell (hUC-MSC) therapy is considered as a promising approach in the treatment of intrauterine adhesions (IUAs). Considerable researches have already detected hUC-MSCs by diverse methods. This paper aims at exploring the quantitative distribution of CM-Dil-labeled hUC-MSCs in different regions of the uterus tissue of the dual injury-induced IUAs in rats and the underlying mechanism of restoration of fertility after implantation of hUC-MSCs in the IUA model.

**Methods:**

In this study, we investigated the quantification of the CM-Dil-labeled hUC-MSCs migrated to the dual injured uterus in Sprague Dawley rats. Additionally, we investigated the differentiation of CM-Dil-labeled hUC-MSCs. The differentiation potential of epithelial cells, vascular endothelial cells, and estrogen receptor (ER) cells were assessed by an immunofluorescence method using CK7, CD31, and ERα. The therapeutic impact of hUC-MSCs in the IUA model was assessed by hematoxylin and eosin, Masson, immunohistochemistry staining, and reproductive function test. Finally, the expression of TGF-β1/Smad3 pathway in uterine tissues was determined by qRT-PCR and Western blotting.

**Results:**

The CM-Dil-labeled cells in the stroma region were significantly higher than those in the superficial myometrium (SM) (71.67 ± 7.98 vs. 60.92 ± 3.96, *p* = 0.005), in the seroma (71.67 ± 7.98 vs. 23.67 ± 8.08, *p* = 0.000) and in the epithelium (71.67 ± 7.98 vs. 4.17 ± 1.19, *p* = 0.000). From the 2nd week of treatment, hUC-MSCs began to differentiate into epithelial cells, vascular endothelial cells, and ER cells. The therapeutic group treated with hUC-MSCs exhibited a significant decrease in fibrosis (TGF-β1/Smad3) as well as a significant increase in vascularization (CD31) compared with the untreated rats.

**Conclusion:**

Our findings suggested that the distribution of the migrated hUC-MSCs in different regions of the uterine tissue was unequal. Most cells were in the stroma and less were in the epithelium of endometrium and gland. Injected hUC-MSCs had a capacity to differentiate into epithelial cells, vascular endothelial cells, and ER cells; increase blood supply; inhibit fibration; and then restore the fertility of the IUA model.

## Background

Intrauterine adhesions (IUAs), also called Asherman’s syndrome, lead to the uterine cavity obliteration and have serious life consequences for women because they cause pelvic pain, menstrual aberrations, recurrent pregnancy loss, and mechanical infertility [1, 2]. The loss of stroma for endometrial impairment caused by the intrauterine operation is believed to be the main cause of IUAs [3], and adhesions formed in where lost stroma is replaced by fibrous tissue [4]. Commonly, hysteroscopic lysis of adhesions prevents adhesion recurrent and improves endometrial regeneration that has been used to treat IUAs, but it is still a significant women’s health challenge for the high recurrence [5]. Recently, some promising new researches suggested that women with IUAs who received human umbilical cord mesenchymal stem cells (hUC-MSCs) therapy can achieve improved fertility outcomes with minimal side-effect [6–8].

HUC-MSCs can be collected easily and non-invasive; no ethical constraints for the umbilical cord is a discarded tissue and hold a high in vitro proliferative rate [9]. Moreover, they are immunoprivileged and can secrete various cytokines, trophic factors, and have strong anti-inflammatory and immunomodulatory properties [10]. For all of the above, hUC-MSCs are an advantageous and beneficial source in regenerative medicine. About mesenchymal stem cell (MSCs) tracking, there are diverse fluorescent dyes that have been used to be orientated in target tissues [11]. Among these, CM-Dil is highly photostable. Despite the fact that the lipophilic dyes heavily bind to the cell membrane phospholipids, there is no toxic photostaining of cells labeled with a low concentration of CM-Dil [12]. Under the excitation of green light, it emits red fluorescence. It is easy and rapid to apply.

HUC-MSCs have exhibited considerable therapeutic potential for IUAs at present. However, the cell mechanism and cell distribution are far from well elucidated. This study aimed to determine the quantitative distribution of labeled hUC-MSCs in different regions of the injured uterine and the underlying mechanism of restoration of fertility after implantation of CM-Dil-labeled hUC-MSCs in a rat IUA model established by dual injury, which is significant for the further study of the treatment of IUAs.

## Methods

### hUC-MSC cultures

Following informed written informed consent from each donor mother, fresh hUCs were collected from term deliveries at an operating room. All clinical treatments involving patients were complied with the Declaration of Helsinki guidelines. This study was approved by the Local Institutional Review Board (registration number: 2019 - P041).

As previously described [13], after rinsing with normal saline (0.9% sodium chloride), the UCs were collected and transferred to the lab in a sterile bottle on ice. The Wharton’s jelly (WJ) was obtained by dissection and removal of the UC arteries, vein, and amniotic epithelium followed by three washes with sterile phosphate-buffered saline (PBS). Subsequently, mince each WJ segment in very small pieces (2–3 mm^3^); transfer them into 10-cm tissue culture dishes (Corning); culture in MSC medium (Beijing Jing-Meng Cell Biological Technology Co., Ltd.) consisting of a serum-free basic medium, serum-free nutritional supplement, and 100,000 U/ml of penicillin/streptomycin; and then maintain in a humidified 5% CO_2_ incubator at 37 °C. After approximately 12 days, the adherent cells were harvested by stem cell digestive juice (Beijing Jing-Meng Cell Biological Technology Co., Ltd.) treatment for subculturing. Cells from passages (P) 2 and 5 were used for the following experiments.

### hUC-MSC morphology and flow cytometry analysis

hUC-MSC morphology was observed by an inverted microscope at P3, and representative pictures were captured at 10×. Moreover, the expression of hUC-MSC-related surface markers such as CD90 and CD105 and the lack of the HLA-DR and CD45 markers were evaluated by flow cytometry. In brief, after reaching 80% confluency, adherent cells were harvested and resuspended in PBS. Aliquots of 1 × 10^6^ cells were incubated with FITC-labeled anti-CD45, anti-CD90, PE-labeled anti-CD34, anti-CD73, APC-labeled anti-CD29, anti-HLA-DR, and PerC-labeled anti-CD105 in the dark at 4 °C for 30 min. After staining, the cells were fixed using paraformaldehyde, and the expression of the cell surface markers was detected using flow cytometry.

### CM-Dil-labeled hUC-MSCs and proliferation assays

Add culture medium containing 2 mg/L CM-Dil (Thermo Fisher Scientific Inc., Waltham, MA, USA) when hUC-MSCs reach 80% confluency after two rinses with sterile PBS. Then incubate for 10 min at 37 °C and 15 min at 4 °C. Next, the labeled cells were monitored for fluorescence using the Olympus BX51 microscope (Olympus, Tokyo, Japan) followed by two washes with sterile PBS. Subsequently, the cells were detached and subcultured. Viability and proliferation of hUC-MSCs and CM-Dil-labeled hUC-MSCs at P3 were analyzed with the MTT method, according to the manufacturer’s instructions. They were seeded in 96-well plates at a concentration of 5 × 10^3^ cells/well in the FBS medium. The assay was performed every 24 h during 7 days. The absorbance was measured at a wavelength of 570 nm by using a spectrophotometric plate reader (Mithras LB 940, Berthold Technology, BadWildbad, Germany).

### Rat IUA model and group

All procedures that involved animals were approved by the Local Institutional Animal Ethics Committee. Our rat model of IUAs was induced as previously described [14, 15]. Briefly, the rats at estrus were operated on through an incision (2–2.5 cm) in the middle line of the abdomen after being anesthetized successfully. Then scrape the endometrial lining by a 2.5-mm endometrial curette (RWD Life Science Co., Ltd., Shenzhen, China). Curettage would not cease until the uterine wall became rough. Subsequently, place the end of the lipopolysaccharide cotton suture into one of the uterine horns cavity, fix the other end on the skin through the muscle layer, and remove the suture after 48 h. Female adult Sprague Dawley(SD) rats (Shijiazhuang, Hebei, China) weighing 220–250 g, aged 10–12 weeks, were randomly assigned to three following groups: (1) normal rat receiving 500 μl PBS via intraperitoneal injection as the normal group (*n* = 15); (2) rat, 2 weeks after surgery, receiving 500 μl PBS via intraperitoneal injection as the model group (*n* = 15); (3) rat, 2 weeks after surgery, receiving 2 × 10^6^ CM-Dil-labeled hUC-MSCs in 500 μl PBS via intraperitoneal injection as the model with the hUC group (*n* = 25). All the study rats were euthanized on the 1st, 2nd, 3rd, and 4th weeks after hUC-MSCs injected.

### In vivo CM-Dil-labeled hUC-MSC tracing

After retrieval of the uteri on the 1st, 2nd, 3rd, and 4th weeks after hUC-MSCs injected, they were embedded in OCT and sectioned (5-μm thick). Representative sections were stained with DAPI to examine the survival and migration of hUC-MSCs in vivo.

### Quantification of CM-Dil-labeled hUC-MSCs

Along the direction perpendicular to the long axis of the uterus, each uterus on the 1st week after hUC-MSCs injected was cut into roughly three equal pieces, then randomly equidistant selected three tissue masses in each rat. Each mass performed ten serial sections (5 μm) after frozen OCT-embedded tissue, then randomly equidistant selected 6 sections [16]. CM-Dil-labeled hUC-MSCs were counted in the 6 sections in each rat of the above groups by a fluorescent microscope (Olympus, Tokyo, Japan). The mean numbers of the fluorescent cells were calculated in the serosa, deep myometrium (DM), superficial myometrium (SM), stroma, and epithelium of the uterus, separately. Western blot was performed to detect the CXC chemokine receptor 4 (CXCR4) to confirm hUC-MSC migration to the target tissue.

### Differentiation of CM-Dil-labeled hUC-MSCs

To assess the differentiation of the cells, the sections obtained from the samples on the 1st, 2nd, 3rd, and 4th weeks after hUC-MSCs injected were washed repeatedly with PBS solution to remove OCT, and then, to reduce non-specific background, sections were treated with 0.3% bovine serum albumin solution in PBS for 30 min. Samples were stained using cytokeratin 7 rabbit polyclonal (CK7, 1:400; Servicebio Technology Co., Ltd., Catalog No. GB11225), CD31 mouse monoclonal (1:200; Servicebio Technology Co., Ltd., Catalog No. GB12063), and estrogen receptor alpha (ERα, 1:200; Biosynthesis Biotechnology Co., Ltd., Catalog No. bs-0725R). The secondary antibody of Alexa Fluor® 488 Goat Anti-Rabbit antibody (1:200; Invitrogen, CA) and Alexa Fluor® 594 Goat Anti-Mouse antibody (1:100; Invitrogen, CA) were used and counterstained with DAPI. Visualization was performed using an optical microscope (Olympus, Tokyo, Japan).

### Evaluation of the effect of hUC-MSC therapy on IUA model

Six weeks post-procedure, the uteri of the three groups were removed, washed with sterile PBS, fixed with 4% buffered formaldehyde, embedded in paraffin, serially sectioned at 5 μm thickness, and routinely stained with hematoxylin and eosin (H&E) and Masson stains. The sections obtained from the samples collected from the above were also used to detect the expression of CD31 (a vascular marker) on the uterine tissues by immunohistochemistry staining.

The function of the regenerative endometrium was evaluated by the reproductive study for a period of 4 weeks. The rats of the three groups were mated with male SD rats. The day of vaginal plug presence was considered gestation day 0. Rats were euthanized at gestation days 15–18, and the presence of embryos were examined.

### Detection of the expression of TGF-β1/Smad3 pathway in uterine tissues

Real-time PCR was performed using a PrimeScript RT reagent kit (Promega) on an Applied Biosystems 7300 Fast Real-Time Polymerase Chain Reaction System. The sequences of the specific primers used were as follows: transforming growth factor-β1(TGF-β1): 5′-ATTCCTGGCGTTACCTTGG-3′(forward), 5′-AGCCCTGTATTCCGTCTCCT-3′(reverse); Smad3: 5′-AGGAGAAGTGGTGCGAGAAG-3′(forward), 5′-GTGACCTGGGGATGGTAATG-3′(reverse). The mRNA expression of each sample on the 2nd and 4th week after hUC-MSC injection was determined after correction by GAPDH expression. The relative expression was calculated using the 2–ΔΔCT method.

The tissues above were quickly removed and homogenized using RIPA buffer and phenylmethylsulfonyl fluoride (Beijing Solarbio Science & Technology Co., Ltd.) by sonication. Proteins from the supernatant were separated by electrophoresed in 10% SDS-PAGE gels and transferred onto polyvinylidene difluoride (PVDF) membranes (Beijing Solarbio Science & Technology Co., Ltd.). The PVDF membranes were incubated with primary antibodies, rabbit polyclonal anti-TGF-β1 (1: 1000, Wanleibio), rabbit monoclonal anti-Smad3 (1: 1000, Wanleibio), and rabbit polyclonal anti-β-actin (1: 1000, Millipore) at 4 °C overnight after being blocked. The membranes were incubated with anti-rabbit IgG (1: 2000, Rockland), a fluorescent labeled IRDye 800 secondary antibody, in the second day. Blots were digitally imaged with a LICOR Odyssey (Lincoln, NE).

### Statistical analysis

Statistical analysis was performed using the Statistical Package for Social Science (SPSS) version 21.0 (IBM Corp, USA). Continuous variables were presented as mean ± standard deviation. One-way ANOVA was used for the homogeneous variance in three or more group comparisons followed by the LSD method between groups. Welch’s ANOVA was applied to the uneven variance in three or more group comparisons followed by the Games-Howell method between groups. A *p* value of < 0.05 was considered to be a significant difference.

## Results

### hUC-MSC characterization and CM-Dil-labeled hUC-MSCs

**Fig. 1:**
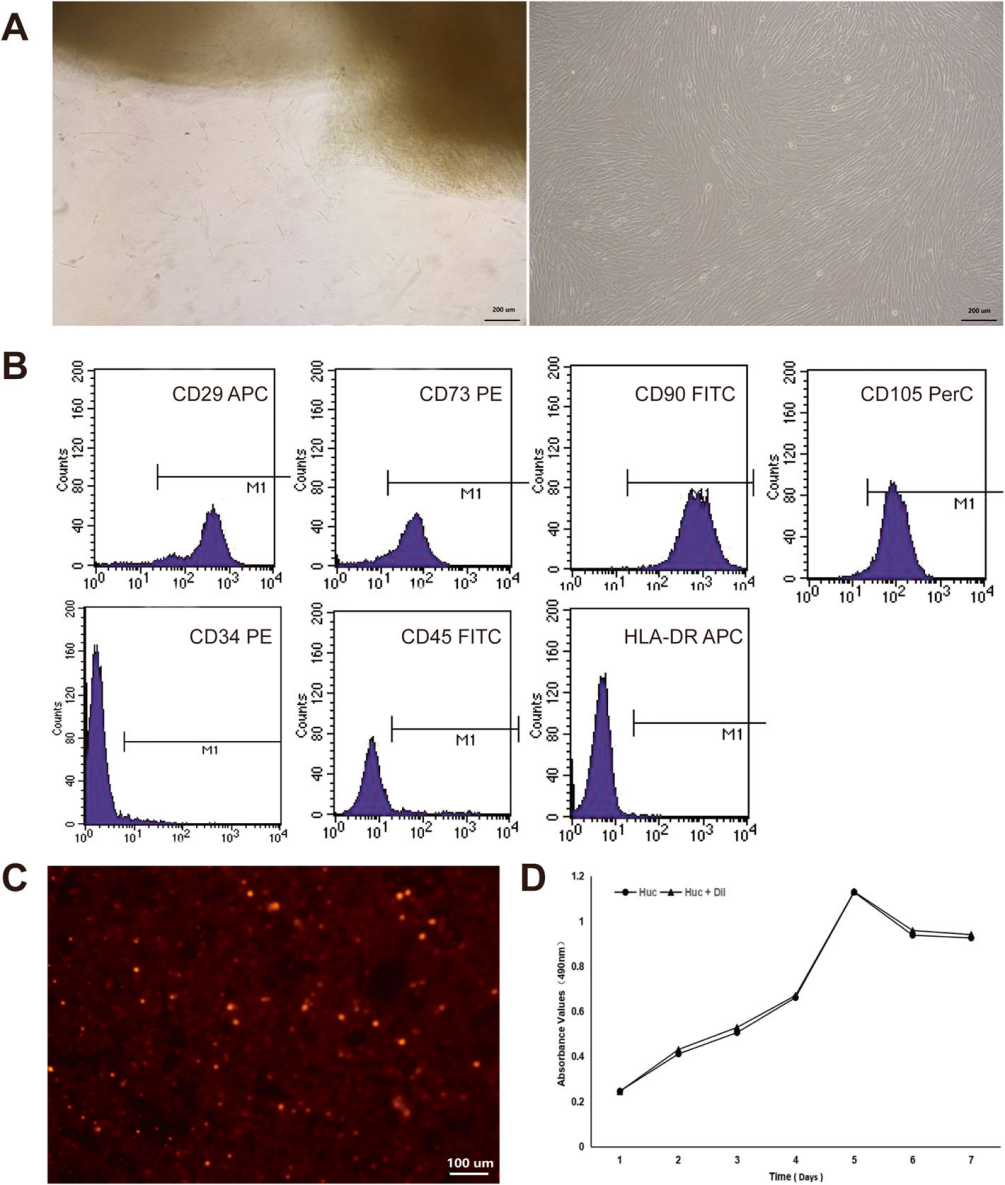
Human umbilical cord mesenchymal stem cells (hUC-MSCs) characterization and CM-Dil-labeled hUC-MSCs (**a**–**d**). After 1–2 days of inoculation, the tissue masses began to adhere to the wall, and 6–7 days after inoculation, the cells could be seen climbing out of the tissue mass, mostly in fusiform shape; the cell fusion reached more than 80% in about 2 weeks, showing a long and fibrous mesenchymal-like morphology, and then following cell passage (**a**). The flow cytometry indicated that CD29, CD73, CD90, and CD105 were positive markers; CD34, CD45, and HLA-DR were the negative markers of hUC-MSCs at passage 3 (**b**). Ninety percent of CM-DiI-labeled cells were observed under a fluorescence microscope (**c**). The stained cells presented the same proliferation rate with hUC-MSCs at passage 3 (**d**)

We isolated hUC-MSCs from WJ explants, the primary cultures of adherent cells with a mesenchymal-like morphology, and were subcultured on average at 12 days after plating. The cells maintained their morphology after subculturing (Fig. 1a). The immunophenotyping of hUC-MSCs at P3 was characterized by flow cytometry, and the results indicated that CD29, CD73, CD90, and CD105 were positive markers; CD34, CD45, and HLA-DR were the negative markers (Fig. 1b). CM-Dil-labeled hUC-MSCs were observed by a fluorescence microscope at the magnification of 100. There were more than 90% of hUC-MSCs labeled with CM-Dil, and the stained cells showed red circular fluorescence (Fig. 1c). Furthermore, it presented the same proliferation rate as hUC-MSCs during the 7 days (Fig. 1d).

### The migration of CM-Dil-labeled hUC-MSCs

**Fig. 2:**
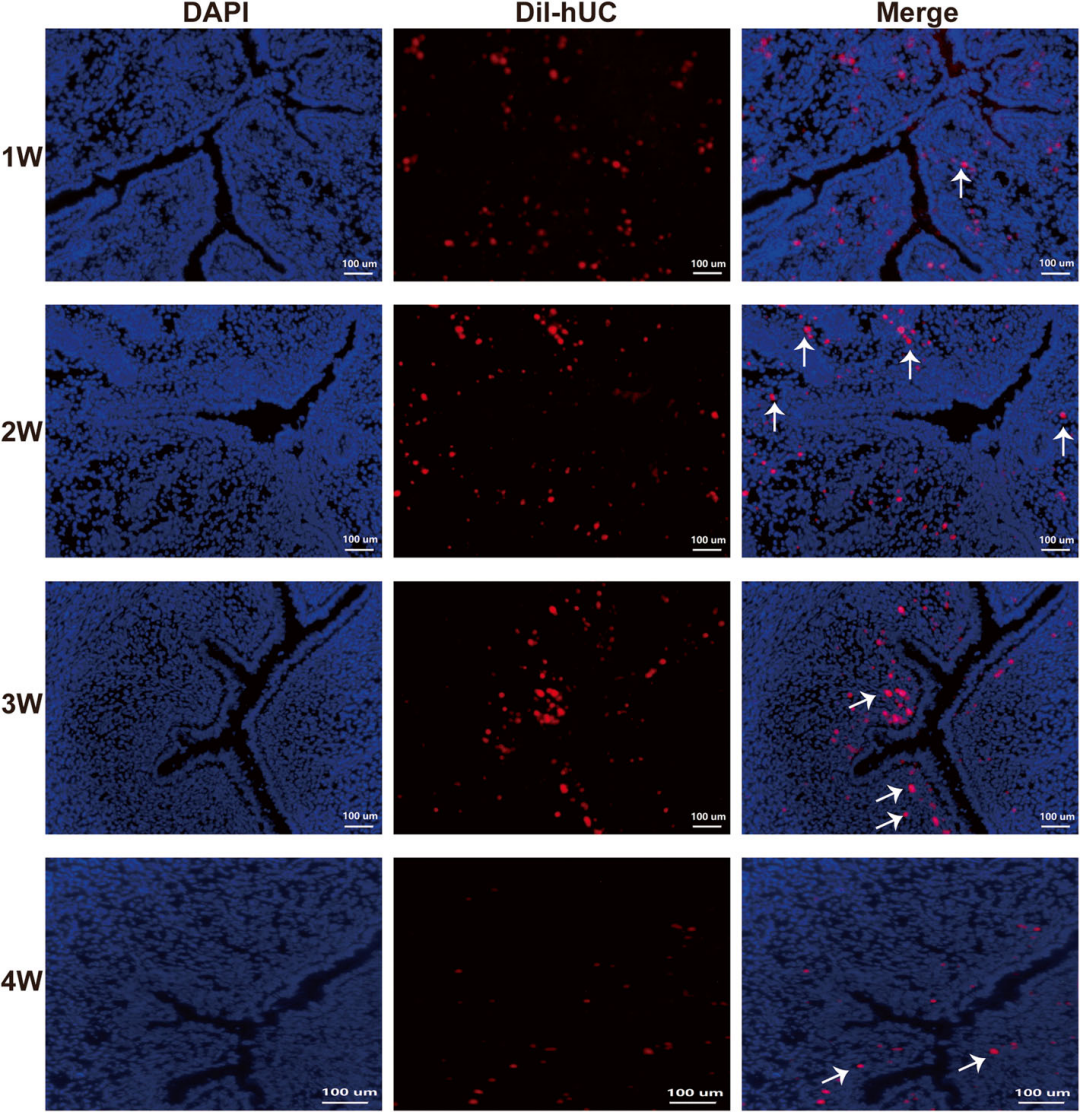
In vivo CM-Dil-labeled hUC-MSC tracing. DAPI located in the nucleus (blue), hUC-MSCs located in the cell membrane and cytoplasm (red), the superposition color of red and blue was magenta representing the living cells, and dead cells were dyed red (arrow). The number of the cells was the most on the 1st week after treatment, and the survival number decreased gradually with the extension of treatment time

The frozen sections were observed under a fluorescence microscope, and red fluorescence was observed in the model with the hUC group, which were the CM-Dil-labeled hUC-MSCs. The number of the cells was the most on the 1st week after treatment, and the survival number decreased gradually with the extension of treatment time (Fig. 2).

### Quantitative assessment of CM-Dil-labeled hUC-MSCs

**Fig. 3:**
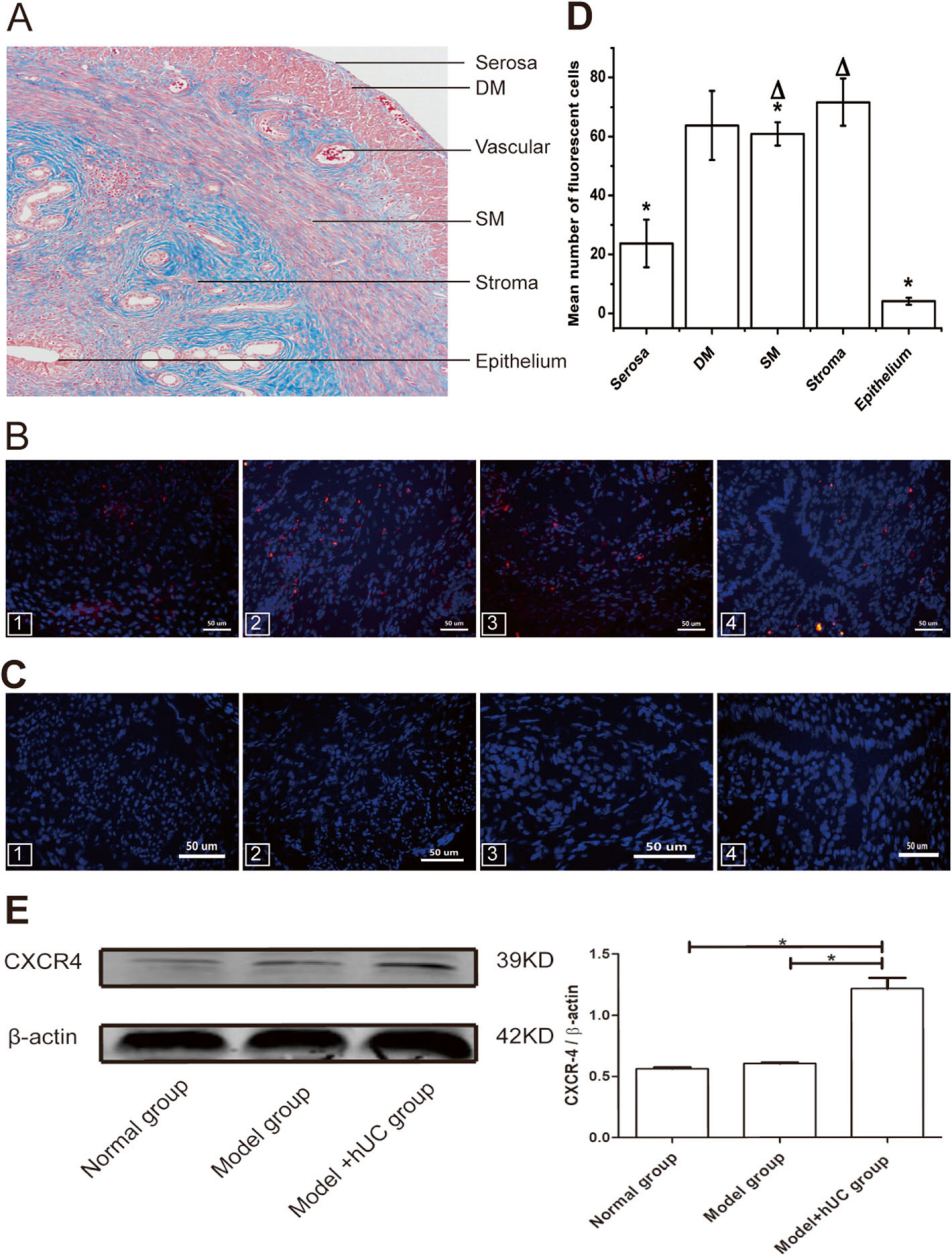
The red fluorescent cells were observed in the rat uterus regions (**a**–**e**). The uterus tissue consists of endometrium epithelium, stroma, deep myometrium (DM), superficial myometrium (SM), and serosa, from the inside to the outside (**a**). CM-Dil-Labeled hUC-MSCs were detected in the serosa and DM (1), in the SM (2), in the stroma (3), but not found in the epithelium of endometrium and gland (4) from the model with hUC group (**b**). The fluorescent cells were not detected in the same regions of the other groups (**c**). The number of fluorescent cells in different regions of the uterus in the model with the hUC group was counted. CM-Dil-labeled cells in the stroma region were significantly higher than those in the seroma (*p = 0.000), in the epithelium (*p = 0.000), and in the SM (*p = 0.005). However, there were no differences between the stroma and the DM (^∆^*p* = 0.93), or the DM and the SM (^**∆**^*p* = 0.33) (**d**). Detection of the CXCR4 expression around the damaged uterine tissues in terms of protein level. The protein level of CXCR4 in the model with the hUC group was significantly higher than those in the other groups. *p < 0.05 was considered to be a significant difference (**e**)

The uterus tissue consists of endometrium epithelium, stroma, SM, DM, and serosa, from the inside to the outside (Fig. 3a). The frozen OCT-embedded uterine tissue sections from the three groups were examined to observe the CM-Dil-labeled hUC-MSCs by a fluorescent microscope. They were detected in different parts of the uterine tissue such as the serosa, DM, SM, and stroma from the model with the hUC group, but the cells were not found in the epithelium of endometrium and gland (Fig. 3b) and in the tissue sections of other groups (Fig. 3c). Welch’s ANOVA was applied to the uneven variance in the five region comparison followed by the Games-Howell method between regions. As shown in Fig. 3d, a significant difference was found in the five regions (*p* = 0.000). The CM-Dil-labeled cells in the stroma region were significantly higher than those in the SM (71.67 ± 7.98 vs. 60.92 ± 3.96, *p* = 0.005), in the seroma (71.67 ± 7.98 vs 23.67 ± 8.08, *p* = 0.000) and in the epithelium (71.67 ± 7.98 vs 4.17 ± 1.19, *p* = 0.000). However, there were no differences between the stroma and the DM (71.67 ± 7.98 vs. 63.75 ± 11.67, *p* = 0.93), or the DM and the SM (63.75 ± 11.67 vs 60.92 ± 3.96, *p* = 0.33).

### Detection of CXCR4 in the uterus tissue

To better confirm hUC-MSC migration to the injury uterus on the 1st week after hUC-MSC injection, CXCR4 expression around the damaged area was examined in terms of protein level. As shown in Fig. 3e, CXCR4 derived from the model with the hUC group was increased, but not in other groups, and a significant difference was found in the three groups (*p* = 0.003) calculated by Welch’s ANOVA. Also, the protein level of CXCR4 in the model with the hUC group was significantly higher than those in the normal group (*p* = 0.01) and the model group (*p* = 0.013) calculated by Games-Howell.

### Differentiation evaluation of CM-Dil-labeled hUC-MSCs

**Fig. 4:**
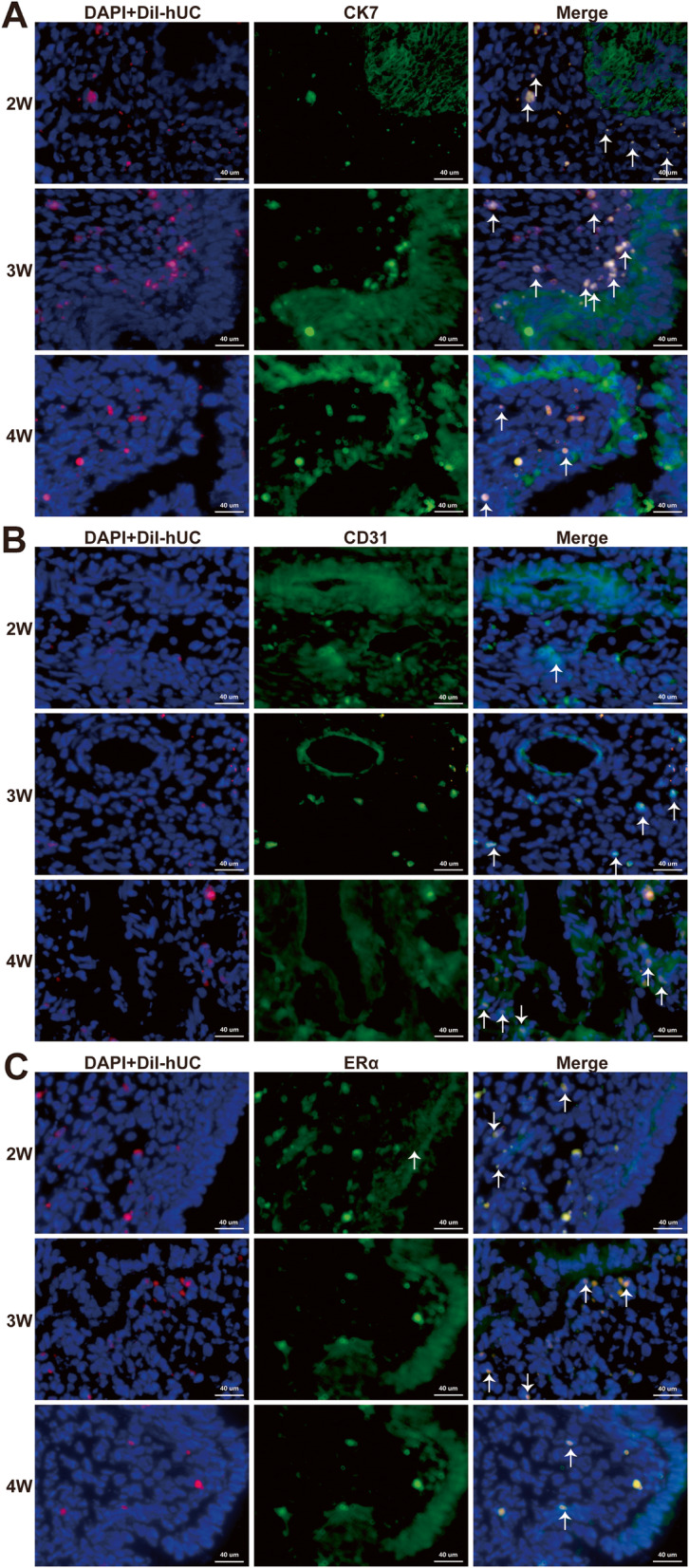
Differentiation evaluation of CM-Dil-labeled hUC-MSCs. The target protein color was green, the DAPI with Dil-hUC color was magenta representing the living cells, and the superposition color of green and magenta was white which means differentiated cells (arrow). CK7 was expressed in the cytoplasm of epithelial cells, CD31 was expressed in the cytoplasm of vascular endothelial cells, and ERα was expressed in the nucleus and cytoplasm. From the 2nd week of treatment, hUC-MSCs began to differentiate into epithelial cells, vascular endothelial cells, and a small amount of ER cells

As Fig. 4 shown that the target protein color was green, the CM-Dil color was red (located in the cell membrane and cytoplasm), DAPI color was blue (located in the nucleus), the superposition color of red and blue was magenta, and the superposition color of green, red, and blue was white. No hUC-MSC differentiation was observed in the 1st week of treatment (Supplementary Fig. 1). From the 2nd week of treatment, hUC-MSCs began to differentiate into epithelial cells and a small amount of ER cells (Fig. 4a, c). With the extension of treatment time, hUC-MSCs and CD31 were in the same position, and some of the co-expressed sites were in the location of vascular endothelial cells (Fig. 4b).

### Evaluation of the effect of hUC-MSC therapy on IUA model

**Fig. 5:**
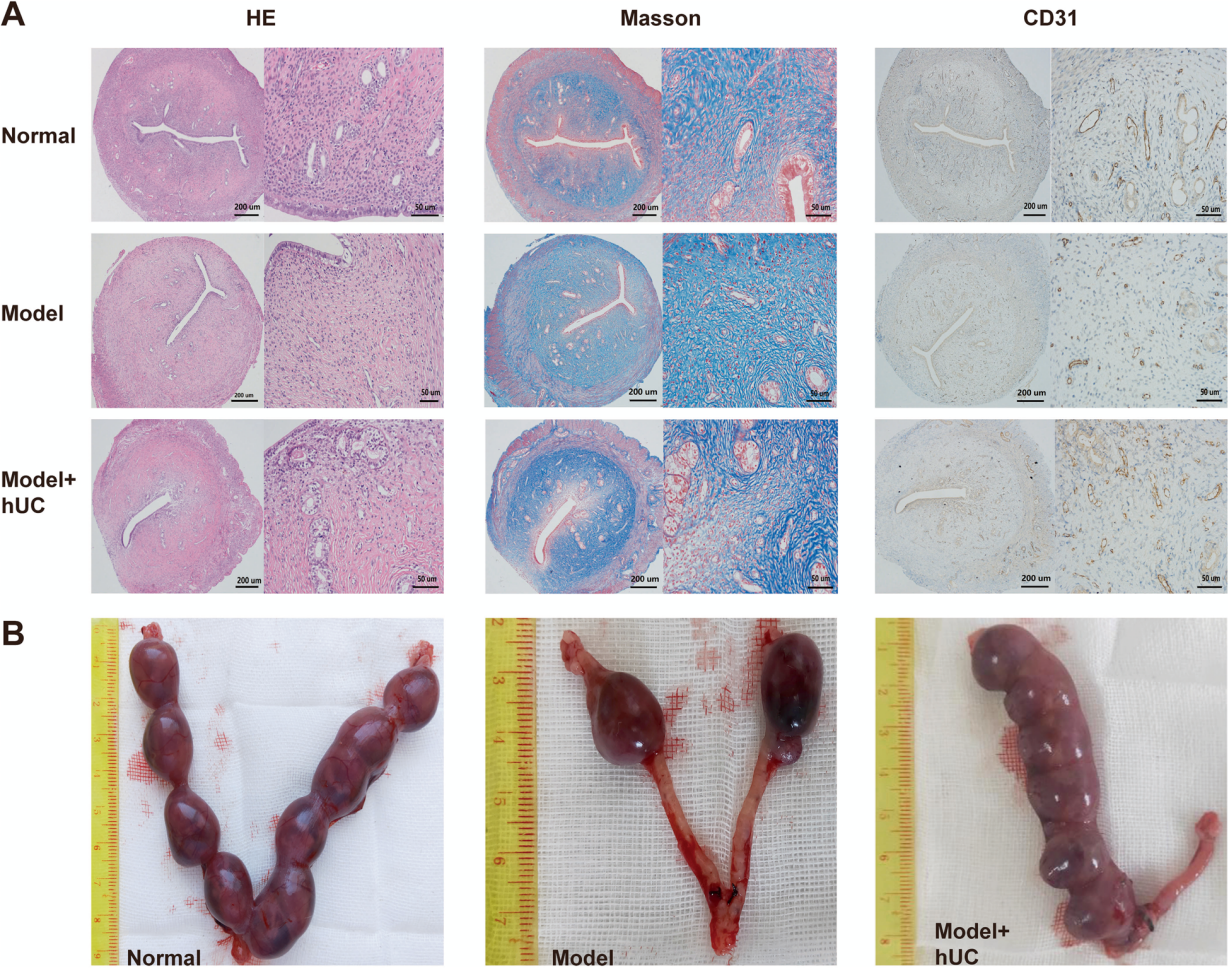
Evaluation of the effect of hUC-MSC therapy on IUA model (**a**–**b**). Photomicrographs represent the three different morphological evaluation methods about the efficacy: HE, Masson, and CD31 staining (**a**). The number of fetuses in the model with the hUC group was more than that in the model group (**b**)

The uterine tissue sections of the three groups on the 6th week post-procedure were used to evaluate the histological changes for confirming the efficacy of hUC-MSCs. H&E, Masson, and CD31 staining showed that after therapy, the endometrial thickness, the number of endometrial glands, and the CD31 positively stained vessels were increased, and the fibrous tissues of the stroma were decreased compared with the model group, and the manifestations were similar in both the normal group and the model with the hUC group (Fig. 5a). The pregnancy outcomes were shown in Fig. 5b, implantation rates were 100% (5 of 5) in the normal group, 20% (1/5) in the model group, and 80% (4 of 5) in the model with the hUC group. The number of fetuses in the model with the hUC group was more than that in the model group (mean 4.25 vs. 1.50).

### Detection of the expression of TGF-β1/Smad3 pathway in uterine tissues

**Fig. 6:**
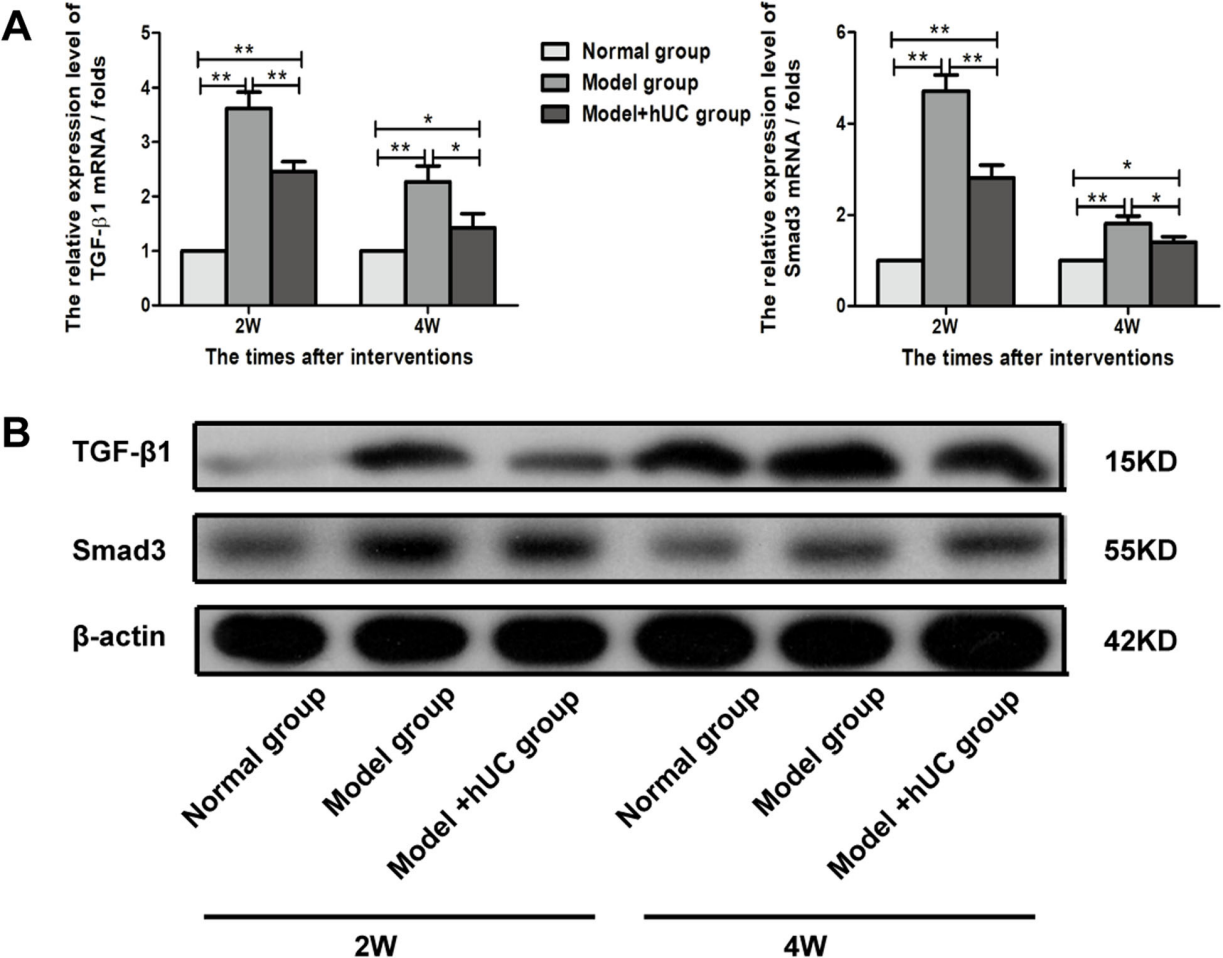
The expression of TGF-β1/Smad3 pathway in uterine tissues in different time points (**a**–**b**). Quantitative analysis for TGF-β1 and Smad3 gene expression on the 2nd and the 4th week after the indicated treatment of IUAs with hUC-MSCs (**a**). Data were shown as mean ± SD, *n* = 4; *p < 0.05, **p < 0.001. Western blot detected TGF-β1 and Smad3 protein expression of different groups on the 2nd and the 4th week after therapy (**b**)

Gene expression of TGF-β1 was significantly downregulated in the model with the hUC group when compared with the expression in the model group on the 2nd and 4th weeks after therapy. Smad3 as a downstream effector of TGF**-**β1 signaling showed the same trends as the expression of TGF-β1 (Fig. 6a). TGF-β1 and Smad3 protein expression levels were both lower in the model with the hUC group than in the model group, and the expressions were normalized relative to the expression of β-actin (Fig. 6b).

## Discussion

At present, MSC-based cell therapy is a promising research for the treatment of various diseases. Among these MSCs, hUC-MSCs are preferred in that they can be collected easily, non-invasive, and no ethical constraints; they can secrete various cytokines and trophic factors; and they are immunoprivileged [9, 10]. About IUAs, the current studies tend to focus on the efficacy of MSCs [17, 18]. However, studies on the distribution of hUC-MSCs in the uterus of IUAs after cell transplantation are far from well elucidated.

In this study, CM-Dil-labeled hUC-MSCs were injected into the IUAs rats and red fluorescent cells were observed in the uterine sections. Jalalie et al. [19] also observed the red fluorescent cells in the ovary tissues of premature ovarian failure mouse 1 week after the injection of CM-Dil-labeled MSCs. In addition, CXCR4 is the primary receptor of stromal cell-derived factor-1 (SDF-1), and the CXCR4/SDF-1 axis has an important role in the migration of the MSCs to the dual injured brain [20]. Our analysis showed that CXCR4 was detected in the uterine tissues of the model with the hUC group by Western blot rather than in other groups. Conceivably, our data determined the migration of hUC-MSCs in the uterus of the dual injured rats. Of note, the number of the cells was the most on the 1st week after treatment, and the survival number decreased gradually with the extension of treatment time, which is supported by previous studies that found MSCs do not stay in the body for a long time and were cleared by the body with the extension of time [21–23].

Therefore, we made a quantitative study on the distribution of CM-Dil-labeled hUC-MSCs on the 1st week after hUC-MSCs injected. The study has shown that CM-Dil-labeled hUC-MSCs were distributed unequally in different parts of the uterine tissue. More of the cells migrated to the stroma and myometrium regions, and almost exclusively no hUC-MSCs reached the epithelium of endometrium and gland. What is the reason for the unequal distribution of the hUC-MSCs? It may be due to the fact that the stroma and myometrium are rich in blood vessels than the epithelium, which is consistent with the study that MSCs were mainly distributed along the blood vessels [24]. Moreover, physical barriers in the uterus tissue may induce the homing instinct of MSCs failed in a particular region of the tissue. Auersperg et al. noted that physical barriers such as basal membranes and cell-cell junction complexes could be related to the small numbers of MSC engraftments in the ovarian germinal epithelium [25]. From this, endometrial epithelium may be with such a physical barrier that causes few migrations of hUC-MSCs to the epithelium.

On the other hand, hUC-MSC is a kind of stem cells. It could colonize in the stromal region without any change. Also, it could differentiate into other kinds of cells under the specific niche. Then, we explored the characteristic of hUC-MSCs after implantation. To our surprise, we found that from the 2nd week of treatment, hUC-MSCs began to differentiate into epithelial cells and a small amount of ER cells. With the extension of treatment time, hUC-MSCs and CD31 were in the same position, and some of the co-expressed sites were in the location of vascular endothelial cells. To our knowledge, the formation of IUAs is relevant to some known reasons. The following are only a few examples:

First, the decrease of ER, ERα, and ERbeta was colocalized in the nuclei of many stromal and glandular epithelial cells [26]. ERα in stromal cells has been shown to be required for epithelial responses to estrogen in the mouse uterus [27]. Such a role is supported by a previous study that showed that E2 elicits epithelial mitogenesis which appeared to be a paracrine event mediated by ER-positive stroma [28]. The injury of normal endometrium in IUAs makes the epithelial cells and interstitial glandular cells rich in ERα disappear; however, the expression of ERα in the basal layer and even SM layer is relatively less, resulting in the trauma site that is not sensitive to estrogen stimulation. Some recent studies have confirmed that the expression of ER in the endometrial tissue of IUAs is significantly decreased [18, 29].

Second is the angiogenesis disorder. The formation of IUAs, to a great extent, is related to intrauterine operation [30]. Once the intrauterine operation is damaged to the basal layer, or even to the muscular layer, the state of ischemia and hypoxia hinders the proliferation of epithelial and interstitial cells, resulting in the obstruction of new angiogenesis. The decrease of the expression of vascular endothelial growth factor (VEGF) or microvessel density (MVD) in the endometrium of IUAs indicates the disturbance of angiogenesis. When blocking VEGF, it will completely inhibit the formation of new blood vessels and delay the repair of the endometrium [31]. Therefore, endometrial repair is partially attributed to angiogenesis [32].

Meanwhile, our results have shown that the endometrial thickness, the number of endometrial glands, and the CD31 positively stained vessels were increased, and the fibrous tissues of the stroma were decreased in the model with the hUC group compared with the model group. And the fertility of the model with the hUC group was improved. Taken together, it seems at least possible that hUC-MSCs might exhibit restoration of meaningful fertility through differentiated into the above three types of cells and that during such migration, the mechanisms could be similar or different to those used for the paracrine effect.

Further, TGF-β1 is the most important factor involved in the formation of fibrosis [33]. Under normal physiological conditions, TGF-β1 can promote the repair and healing of the injured site, making the extracellular matrix in a state of balance, while under pathological conditions, TGF-β1 can stimulate the expression of fibronectin and collagen, inhibit the degradation of the extracellular matrix, and lead to fibrosis and scar tissue formation [33]. Plenty of studies have confirmed that TGF-β1/Smad pathway dysregulation was an important mechanism in tissue fibrosis [34–36]. Smad2 and Smad3 are the major downstream regulators and promote TGF-β1 mediate the expression of key tissue fibrosis genes, while Smad7 serves as a negative feedback regulator of the pathway, thereby preventing TGF-β1-mediated fibrosis [37, 38]. Smad3 appeared to be a key element responsible for fibrosis in hepatic fibrosis [39, 40]. And it has been demonstrated that downregulating the expression of TGF-β1/Smad3 by microRNA can reduce endometrial interstitial fibrosis of patients with IUA [35]. Then, we detected the expression of the TGF-β1/Smad3 pathway in uterine tissues and our results are also consistent with the reports [34–36].

To note, TGF-β is thought to have both pro- and anti-angiogenic properties, depending on the levels present. Low levels of TGF-β contribute to angiogenesis by upregulating angiogenic factors and proteases, while high doses of TGF-β stimulate basement membrane reformation, recruit smooth muscle cells, increase differentiation, and inhibit endothelial cell growth [41]. It is conceivable that hUC-MSCs may go through the TGF-β1/Smad3 pathway to regulate uterine neovascularization. Care should be taken when extending our results to other study, however, as the differences between mouse and human uterine epithelia with respect to the above mechanisms may exist.

## Conclusion

In summary, this study shows that CM-Dil-labeled hUC-MSCs home in the dual injured rat uterus with most cells in the stroma and less in the epithelium of endometrium and gland. Injected hUC-MSCs have a capacity to differentiate into epithelial cells, vascular endothelial cells, and ER cells; increase blood supply and inhibit fiber formation; and then restore the fertility of the IUA model. A better understanding of how the hUC-MSC response to the uterine tissues may be beneficial for the further study of the treatment of IUAs.

## Supplementary information

**Additional file 1: Figure S1.** There was no hUC-MSCs differentiation on the 1sth week after hUC-MSCs injected.

## Data Availability

Not applicable.

## Abbreviations

IUAs: Intrauterine adhesions
hUC-MSCs: Human umbilical cord mesenchymal stem cells
MSCs: Mesenchymal stem cells
WJ: Wharton’s jelly
PBS: Phosphate-buffered saline
P: Passages
SD: Sprague Dawley
DM: Deep myometrium
SM: Superficial myometrium
CXCR4: CXC chemokine receptor 4
CK7: Cytokeratin 7
ERα: Estrogen receptor alpha
H&E: Hematoxylin and eosin
TGF-β1: Transforming growth factor-β1
PVDF: Polyvinylidene difluoride
SPSS: Statistical package for social science
SDF-1: Stromal cell-derived factor-1
MVD: Microvessel density
VEGF: Vascular endothelial growth factor
