# Supplementary figures and images for: Quantification of the CM-Dil-labeled human umbilical cord mesenchymal stem cells migrated to the dual injured uterus in SD rat
Source: Stem Cell Res Ther. 2020 Jul 13;11:280. doi: 10.1186/s13287-020-01806-4 (PMC7359016; doi:10.1186/s13287-020-01806-4)

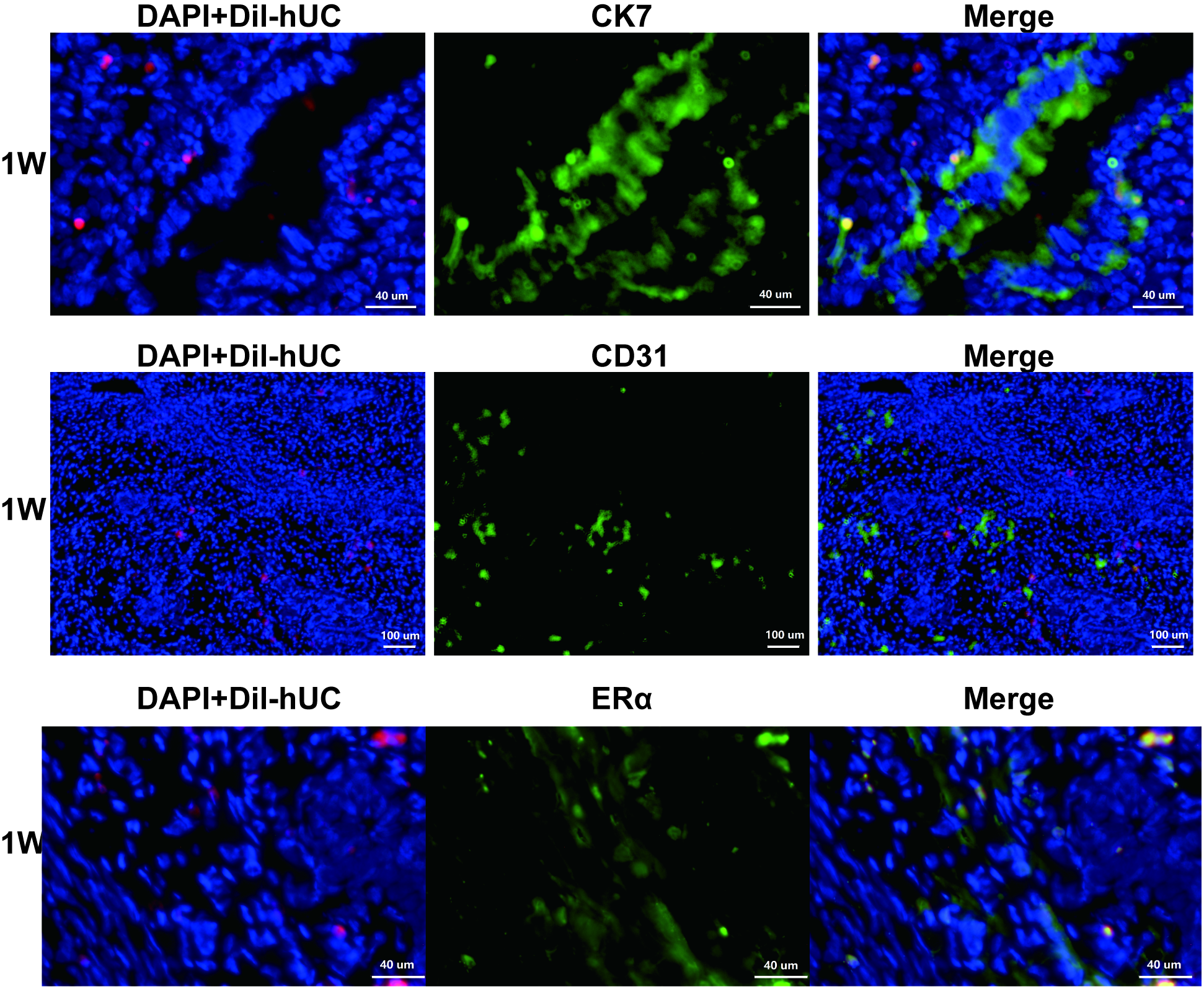

Supplement: Supplementary file 1 — Additional file 1: Figure S1. There was no hUC-MSCs differentiation on the 1sth week after hUC-MSCs injected. [file 13287_2020_1806_MOESM1_ESM.tif]
